# Complete Genome Sequence of Aeromonas rivipollensis KN-Mc-11N1, Isolated from a Wild Nutria (Myocastor coypus) in South Korea

**DOI:** 10.1128/MRA.00907-18

**Published:** 2018-08-02

**Authors:** Seon Young Park, Se Ra Lim, Jee Soo Son, Hye Kwon Kim, Sun-Woo Yoon, Dae Gwin Jeong, Moo-Seung Lee, Jung Ro Lee, Do-Hun Lee, Ji Hyung Kim

**Affiliations:** a Infectious Disease Research Center, Korea Research Institute of Bioscience and Biotechnology, Daejeon, Republic of Korea; b Department of Animal Science, College of Life Sciences, Pusan National University, Miryang, Republic of Korea; c Bio-analytical Science Division, University of Science and Technology (UST), Daejeon, Republic of Korea; d iNtRON Biotechnology, Inc., Seongnam, Republic of Korea; e Division of Ecological Conservation Research, National Institute of Ecology, Seocheon, Republic of Korea; University of Arizona

## Abstract

We report here the complete genome sequence of Aeromonas rivipollensis KN-Mc-11N1, which was isolated from a wild nutria (Myocastor coypus) in South Korea. Genomic analysis indicated that A. rivipollensis may have zoonotic potential similar to that of other aeromonads, and nutria could be one of the sources of transmission of zoonotic pathogens to humans.

## ANNOUNCEMENT

Although the genus Aeromonas is ubiquitous in aquatic environments, several species of the genus have been recognized as pathogens of cold-blooded animals (1). Interests in this genus have increased due to its zoonotic potential and the emergence of antibiotic resistance (2), and recent studies have indicated that domestic and wild animals could be the sources of transmission of aeromonads to humans (3–6). Since 2016, we have investigated the occurrence of potential zoonotic aeromonads in animals to implement the one-health perspective on emerging public health threats. Herein, we present a complete genome sequence of A. rivipollensis, which is the first genome sequence from a representative of this species.

The strain KN-Mc-11N1 was isolated from the nasal swab of an adult female wild nutria (Myocastor coypus) carcass collected at the Nakdong River (South Korea) in June 2017 using standard dilution plating on sheep blood agar (Hanil Komed), followed by incubation at 37°C. The β-hemolytic and cefoxitin-susceptible isolate was identified to be a member of the genus Aeromonas based on biochemical and 16S rRNA analyses, and the *gyrB* (98.0%) and *rpoB* (99.2%) genes of the isolate were most similar to those of A. rivipollensis P2G1^T^, among the available species of the genus. Therefore, the isolate was classified as A. rivipollensis following its emended species description (7).

Although A. rivipollensis was recently described (8, 9), its taxonomical relatedness to the Aeromonas media species complex has not been fully understood due to the absence of a genome sequence (8, 10). Therefore, the genome of KN-Mc-11N1 was sequenced using a hybrid approach with a PacBio RS II system (Pacific Biosciences) and HiSeq 2000 platform (Illumina). The PacBio reads (1,643,982,719 bp, 155,756 reads, 365× coverage) were assembled with HGAP (version 3.0), and the Illumina paired-end reads (3,474,295,580 bp, 23,008,580 reads, 771× coverage) were mapped using BWA-MEM (version 0.7.15); errors were corrected using Pilon (version 1.21) with default parameters. Genome annotation was performed using the NCBI Prokaryotic Genome Annotation Pipeline (11).

The KN-Mc-11N1 genome was 4,508,901 bp long (86.3% coding regions) with 61.9% G+C content, encoding 4,025 coding sequences (CDS), 31 rRNAs, 124 tRNAs, and 6 noncoding RNAs. The genome similarities of KN-Mc-11N1 and other strains in the A. media species complex were assessed using the ANI Calculator (12), and the genome was most similar to those of the clade A strains (>95%) rather than to that of A. media CECT4232^T^ (93.2%), thus supporting the recent taxonomical revision (7).

Potential virulence-related and antibiotic resistance genes in KN-Mc-11N1 were identified, as previously described (13). Consequently, several genes involved in type II and IV secretion systems and proteolytic and hemolytic activities, which were homologous to those of other pathogenic Aeromonas species, were detected. Moreover, the genome possessed genes involving β-lactam resistance (*bla*_OXA-12_ and *bla*_MOX-1_) and phenicol resistance (*catB10*). These results indicate that A. rivipollensis may have zoonotic potential similar to that of other aeromonads, and nutria could be one of the sources of its transmission to humans.

### Data availability.

A. rivipollensis KN-Mc-11N1 has been deposited in the Korean Culture Center of Microorganisms as KCCM 90285. The KN-Mc-11N1 genome sequence has been deposited at DDBJ/ENA/GenBank under accession number CP027856.
